# Morphometric Analysis of the Openings in the Posterior Cranial Fossa and Their Relationship with Sex

**DOI:** 10.3390/diagnostics15243189

**Published:** 2025-12-13

**Authors:** Ahmet Depreli, Necati Emre Sahin, Sefa Sonmez, Merve Nur Ozgen Sonmez, Mensure Sahin, Berna Dogan, Sadik Bugrahan Simsek, Huseyin Ugur Bakan

**Affiliations:** 1 Morgue Specialization Department, Konya Group Presidency, Konya Council of Forensic Medicine, Konya 42005, Türkiye; ahmetdep@gmail.com; 2 Department of Anatomy, Faculty of Medicine, Karabuk University, Karabuk 78000, Türkiye; mensuresahin@karabuk.edu.tr; 3 Department of Anatomy, Faculty of Medicine, Tokat Gaziosmanpasa University, Tokat 60100, Türkiye; sefa.sonmez@gop.edu.tr (S.S.); mervenur.ozgen@gop.edu.tr (M.N.O.S.); berna.dogan@gop.edu.tr (B.D.); sadik.simsek@gop.edu.tr (S.B.S.); 4 Tokat Branch Office, Tokat Council of Forensic Medicine, Tokat 60100, Türkiye; hbakan1993@yandex.com

**Keywords:** posterior cranial fossa, foramen magnum, jugular foramen, internal acoustic opening, anatomical landmarks, morphometry, sex estimation

## Abstract

**Background/Objectives**: The cranial base, especially the posterior cranial fossa, has openings with population-specific morphometry. This study aimed to assess the morphometric characteristics of the major posterior cranial fossa openings (foramen magnum, jugular foramen, internal acoustic opening) in the Turkish population and evaluate their utility for sex estimation. It also aimed to provide population-specific reference values for forensic anthropology and cranial base surgery. **Methods**: This prospective study included 304 adult skulls (151 female, 153 male) obtained from forensic autopsy cases, all of which had preserved anatomical integrity. Structures in the posterior cranial fossa were exposed following a standardized dissection protocol. A total of 18 morphometric parameters were measured using a digital caliper. Inter-sex comparisons were performed, and the diagnostic performance of the parameters for sex differentiation was evaluated using receiver operating characteristic (ROC) curve analysis. **Results**: All morphometric parameters and inter-foraminal distances were significantly larger in male individuals compared to females (*p* < 0.001). Similarly, ellipticity indices were higher in males than in females (all *p* < 0.001). ROC analysis revealed that right internal acoustic opening transverse diameter (RIAO-T), left and right jugular foramen transverse diameters (RJF-T and LJF-T) parameters possess exceptionally high discriminatory power, yielding accuracies greater than 99%. **Conclusions**: Components of the posterior cranial fossa exhibit marked sexual dimorphism in the Turkish population. These morphometric data provide valuable anatomical references for forensic identification, aid in preserving neurovascular structures, and support safe surgical planning in cranial base procedures.

## 1. Introduction

Identification of human skeletal remains is a central objective in forensic medicine and anthropology. Once the human origin of skeletal material is confirmed, the identification process typically involves six key steps: estimation of the individual’s sex, biological age, stature, ancestry-related traits, postmortem interval, and potential cause of death [1]. Among these, accurate estimation of sex is critically important, as most other parameters rely on sex-specific standards [2].

Morphometric analysis of bones has been shown to provide high accuracy for sex estimation [3]. The reliability of predictions increases with the number of bones and the diversity of parameters analyzed. However, the infrequent availability of complete and well-preserved skeletons necessitates the ability to estimate sex from individual bones or even bone fragments [4]. The skull and pelvis are widely regarded as the most reliable anatomical structures for sex estimation due to their consistently high accuracy, although recent studies have shown that long bones can also achieve high discriminatory performance [5,6]. The skull, with its dense and thick cortical bone, exhibits strong resistance to environmental factors and is therefore frequently well-preserved in both archaeological and forensic contexts [7]. Previous studies have utilized metric measurements of the foramen magnum (FM) and internal acoustic opening (IAO) for sex estimation [8,9,10,11,12]. Similarly, sex estimation based on jugular foramen (JF) measurements has also been reported in the literature [5,13]. A critical emphasis of these studies is that cranial morphometry may vary significantly between populations, highlighting the need for population-specific reference data [14].

A review of the literature indicates that, to date, no study has comprehensively analyzed all openings forming the posterior cranial fossa (FM, JF, and IAO) in the Turkish population while simultaneously evaluating their relationship with sex. This study aims to fill this gap. Morphometric assessment of the posterior cranial fossa also has significant clinical relevance [15]. The cranial base represents a crucial intersection for disciplines such as neurosurgery, otolaryngology, and radiology, particularly in the surgical management of pathologies such as schwannomas, meningiomas, and glomus jugulare tumors. Surgical interventions in this region often require partial petrosectomy, including the jugular fossa [15,16]. Therefore, detailed anatomical knowledge of cranial base openings and the distances between them plays a critical role in surgical planning, guiding the surgeon to minimize the risk of iatrogenic injury to neurovascular structures.

This study aimed to establish a detailed morphometric profile of the major anatomical openings of the posterior cranial fossa FM, JF, and IAO in the Turkish population. The measurements obtained were analyzed comprehensively to assess their discriminative value for sex estimation and their potential contribution to forensic anthropological identification. Additionally, population-specific morphometric data were intended to provide reliable reference values to support preoperative planning in cranial base surgery.

## 2. Materials and Methods

Ethical approval for this prospective study was obtained from the Tokat Gaziosmanpasa University Clinical Research Ethics Committee (Decision No: 24-KAEK-197, Date: 27 June 2024) and the Council of Forensic Medicine (Decision No: 21589509/2024/695, Date: 2 July 2024). Morphometric measurements were performed on adult skulls obtained from forensic autopsy cases. All measurements were recorded systematically and consecutively in an Excel spreadsheet, following a standardized protocol.

### 2.1. Power Analysis

Based on the findings of Tellioglu et al. [17] in a study on the Turkish population, a sample size calculation was performed for the anteroposterior diameter of the FM (FM-AP). Assuming a significance level (α) of 0.05, a statistical power (1–β) of 0.80, an effect size of 0.71, and a two-tailed alternative hypothesis (H_1_), it was determined that a minimum of 32 cases per sex group (a total of 64 cases) was required to detect a significant difference. Sample size calculations were conducted using G*Power 3.1 (Heinrich-Heine-Universität Düsseldorf, Düsseldorf, Germany).

### 2.2. Study Population

A total of 304 cranial base specimens (151 female, 153 male) from forensic autopsy cases were included in the study. The inclusion criteria were defined as adult individuals aged 18 years or older with anatomically intact cranial base structures, without deformity, trauma, or pathological findings, and with verifiable sex information through forensic records. Exclusion criteria included trauma, congenital anomalies, pathological deformities, evidence of surgical intervention, or fractures/structural defects that could interfere with accurate measurements.

Head circumference was measured for all included cases to assess the potential effect of individual cranial size differences, based on the glabella–inion line, on morphometric measurements. Descriptive demographic data of the cases are presented in Table 1.

### 2.3. Dissection Procedure

**Figure 1:**
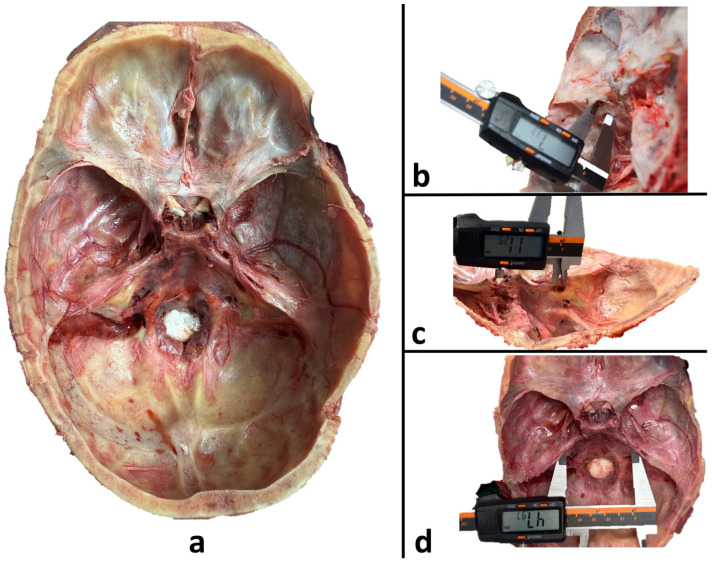
Dissection view of the posterior cranial fossa and morphometric measurements: (**a**) Superior view of the skull base after dissection showing the posterior cranial fossa; (**b**) Measurement of the distance between the left jugular foramen and internal acoustic opening (LJIAO) using a digital caliper; (**c**) Measurement of the anteroposterior diameter of the internal acoustic opening (LIAO-AP); (**d**) Measurements of the intermeatal distance between the right and left internal acoustic openings (II).

Following standard autopsy protocols, a transverse incision was made approximately 1 cm above the glabella extending to the external occipital protuberance, and the SCALP was reflected. The calvaria was removed in the transverse plane using a cranial saw. After separating the dura mater from the bone, meningeal remnants were carefully cleaned. Subsequently, the brain was removed via an incision at the level of the medulla oblongata, aligned with the FM. After these procedures, the inner surface of the occipital bone, including the FM, JF, and IAO, became fully visible. All vessels, nerves, and soft tissue remnants passing through these openings were removed. The bony margins were preserved and cleaned while maintaining anatomical integrity to allow for accurate measurements (Figure 1a).

### 2.4. Measurement Parameters

Morphometric measurements were obtained from the main openings of the posterior cranial fossa and the distances between them. The anatomical landmarks points of the measured openings are presented in Table 2. Measurements were performed using a Valkyrie digital caliper with a precision of 0.01 mm. All measurements were repeated two times by the same investigator, and the mean values were used for analysis.

The FM-AP was defined as the longest linear distance between the basion and opisthion, and the transverse diameter (FM-T) was defined as the widest mediolateral distance. The ellipticity ratio (FM-E) was calculated from these two measurements using the following formula:

(1) Foramen Magnum Ellipticity Ratio = FM-E = (FM-T)/(FM-AP).

The JFs were evaluated separately on the right and left sides. For each JF, the anteroposterior diameter (RJF-AP, LJF-AP) was measured along the anteroposterior axis, and the transverse diameter (RJF-T, LJF-T) along the mediolateral axis. The ellipticity ratio for each JF was calculated as:

(2) Jugular Foramen Ellipticity Ratio = JF-E = (JF-T)/(JF-AP).

For the IAOs, measurements were performed on the right and left sides. The anteroposterior diameter (RIAO-AP, LIAO-AP) representing the anterior–posterior length, and the transverse diameter (RIAO-T, LIAO-T) representing the mediolateral width were recorded (Figure 1c). The ellipticity ratio for each IAO was calculated as:

(3) Internal Acoustic Opening Ellipticity Ratio = IAO-E = (IAO-T)/(IAO-AP).

Distances between openings were also recorded as follows: the distance between the centers of the right and left JF (JJ), the distance between each JF and the corresponding IAO on the same side (LJIAO/RJIAO) (Figure 1b), the distance from each JF to the anterior edge of the FM (basion) (LJFM/RJFM), the distance from each IAO to the FM (LIAOF/RIAOF), and the distance between the centers of the right and left IAO (II) (Figure 1d).

### 2.5. Statistical Analyses

All statistical analyses were performed using R software (version 4.3.3). Since both sex groups included more than 30 participants, parametric testing was considered appropriate in accordance with the Central Limit Theorem, and comparisons between sexes were conducted using the independent samples *t*-test. Descriptive statistics were presented as mean ± standard deviation (SD), median, and minimum (Min)–maximum (Max) values. Intra-observer measurement reliability was examined using the technical error of measurement (TEM), relative TEM (rTEM), and the reliability coefficient (R). These indices were calculated for each morphometric parameter to assess the stability of repeated measurements performed by the same observer.

The diagnostic performance of the morphometric parameters in sex estimation was evaluated using receiver operating characteristic (ROC) analysis within a 10-fold cross-validation framework. In this approach, all performance metrics such as area under the curve (AUC), accuracy, sensitivity, specificity, and bias were calculated separately within each fold using only the test data for that fold. Accuracy in each fold was defined as the proportion of correctly classified individuals, computed as (true positives + true negatives) divided by the total number of cases. Sensitivity represented the proportion of correctly classified females, whereas specificity represented the proportion of correctly classified males, based on the predefined coding of the outcome variable. Bias was calculated as the difference between female and male accuracy within each fold. The final reported values for all metrics correspond to the mean and standard deviation obtained across the 10 cross-validation folds. A *p*-value of <0.05 was considered statistically significant.

## 3. Results

The mean age of the individuals included in the study was 54.56 ± 20.69 years in females and 51.74 ± 21.49 years in males, with no significant difference in age between the sexes (*p* = 0.244). However, males had significantly greater height (172.65 ± 11.03 cm) and head circumference (56.85 ± 1.66 cm) compared with females (both *p* < 0.001) (Table 1).

### 3.1. Measurement Reliability of Morphometric Parameters

Measurement precision indicators for all morphometric parameters are presented in Table 3. TEM values ranged between 0.001 and 0.007, while rTEM values remained below 0.07%. The reliability coefficients (R) varied from 0.965 to 0.991 across parameters.

### 3.2. Morphometric Measurements

The morphometric measurements of the apertures located in the posterior cranial fossa are presented in Figure 1. All linear parameters were significantly higher in males than in females (all *p* < 0.001) (Table 4). FM-AP measured 42.68 ± 2.40 mm in males and 34.45 ± 3.71 mm in females, while FM-T measured 41.34 ± 2.27 mm in males and 32.64 ± 3.18 mm in females. RJF-AP and LJF-AP values were 16.21 ± 1.18 mm and 14.27 ± 1.57 mm in males, and 10.20 ± 2.06 mm and 8.85 ± 1.93 mm in females, respectively. RJF-T and LJF-T measured 9.36 ± 0.67 mm and 8.44 ± 0.65 mm in males, and 5.04 ± 1.35 mm and 4.65 ± 1.28 mm in females. RIAO-AP and LIAO-AP values were 14.84 ± 1.20 mm and 12.97 ± 1.53 mm in males, and 8.93 ± 2.02 mm and 7.56 ± 1.89 mm in females, respectively. RIAO-T and LIAO-T were 9.23 ± 0.66 mm and 8.31 ± 0.84 mm in males, and 4.85 ± 1.34 mm and 4.55 ± 1.40 mm in females. The distance between the centers of the jugular foramina (JJ) was 46.88 ± 2.90 mm in males and 39.21 ± 3.55 mm in females. RJIAO and LJIAO distances were 7.43 ± 1.79 mm and 7.32 ± 1.84 mm in males, and 5.84 ± 1.67 mm and 5.75 ± 1.67 mm in females, respectively. RIAOF and LIAOF measured 18.40 ± 1.73 mm and 17.66 ± 1.82 mm in males, and 16.35 ± 2.03 mm and 15.79 ± 2.12 mm in females. RJFM and LJFM were 17.48 ± 2.03 mm and 17.04 ± 2.01 mm in males, and 15.59 ± 2.24 mm and 15.21 ± 2.16 mm in females. The II was 57.33 ± 5.38 mm in males and 44.50 ± 4.20 mm in females.

### 3.3. Circularity (Ellipticity) Analysis

Ellipticity values were calculated for all apertures. Ellipticity values were higher in males than in females (*p* < 0.001). For the FM, ellipticity measured 0.97 ± 0.03 in males and 0.92 ± 0.02 in females. For the RJF, values were 0.58 ± 0.05 in males and 0.49 ± 0.07 in females. For the LJF, ellipticity measured 0.60 ± 0.08 in males and 0.52 ± 0.07 in females. RIAO ellipticity was 0.62 ± 0.06 in males and 0.55 ± 0.08 in females, while LIAO values were 0.61 ± 0.07 in males and 0.54 ± 0.08 in females. The findings related to the circularity analysis are presented in Table 5.

### 3.4. ROC Analysis for Sex Discrimination

**Figure 2:**
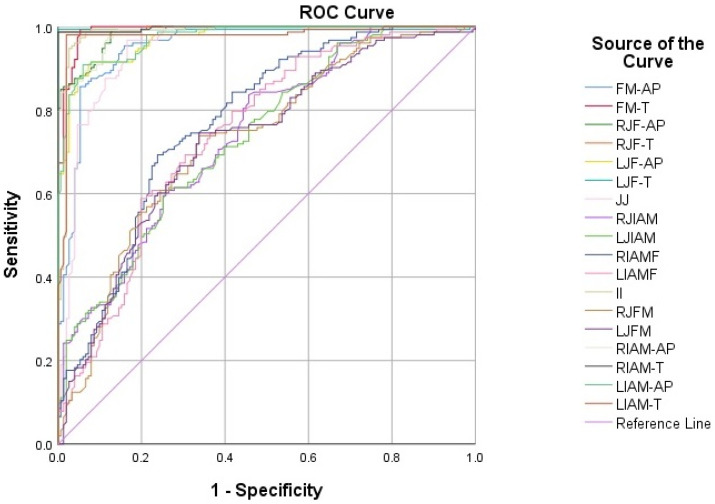
ROC curves showing the discriminative power of morphometric parameters of the posterior cranial fossa for sex differentiation. FM-AP, foramen magnum anteroposterior diameter; FM-T, foramen magnum transverse diameter; RJF-AP, right jugular foramen anteroposterior diameter; RJF-T, right jugular foramen transverse diameter; LJF-AP, left jugular foramen anteroposterior diameter; LJF-T, left jugular foramen transverse diameter; RIAO-AP, right internal acoustic opening anteroposterior diameter; RIAO-T, right internal acoustic opening transverse diameter; LIAO-AP, left internal acoustic opening anteroposterior diameter; LIAO-T, left internal acoustic opening transverse diameter; JJ, distance between right and left jugular foramen; RJIAO/LJIAO, distance between jugular foramen and internal acoustic opening on the right/left; RIAOF/LIAOF, distance between internal acoustic opening and foramen magnum on the right/left. II, intermeatal distance; RJFM/LJFM, distance between jugular foramen and foramen magnum on the right/left.

ROC analysis was performed to evaluate the diagnostic power of morphometric measurements of the posterior cranial fossa in sex discrimination. According to the results presented in Table 6, the parameters with the highest discriminatory power were RJF-T (AUC = 0.998; Accuracy = 0.993), RIAO-T (AUC = 0.998; Accuracy = 0.993), FM-T (AUC = 0.992; Accuracy = 0.971), LJF-T (AUC = 0.992; Accuracy = 0.990), and II (AUC = 0.984; Accuracy = 0.947). However, several parameters demonstrated more limited discriminatory capacity. RIAOF (AUC = 0.791; Accuracy = 0.717), LIAOF (AUC = 0.754; Accuracy = 0.658), RJIAO (AUC = 0.752; Accuracy = 0.691), LJIAO (AUC = 0.743; Accuracy = 0.674), and RJFM (AUC = 0.740; Accuracy = 0.697) exhibited lower discriminative performance for sex estimation (Figure 2) (Table 6).

## 4. Discussion

Sex estimation, a fundamental step in the identification process in forensic anthropology, is the central focus of the present study. In cases where incomplete or fragmentary skeletal remains are encountered, the skull—owing to its robust structure—serves as a critical source of information. This study represents the first comprehensive investigation in the Turkish population to evaluate the role of all major openings of the posterior cranial fossa (FM, JF, and IAO) in sex estimation, focusing on one of the most structurally resilient regions of the cranial base. Our findings demonstrate that both the linear dimensions (distance and diameter) and the shape-related indicator, ellipticity ratio, of these structures exhibit pronounced sexual dimorphism. Furthermore, ROC analysis revealed that RIAO-T, RJF-T, and LJF-T parameters possess exceptionally high discriminatory power, yielding accuracies greater than 99%. Taken together, these results strongly support the potential of posterior cranial fossa morphometry as a reliable and highly sensitive complementary tool in forensic identification protocols.

Our findings clearly indicate that the FM displays marked sexual dimorphism. Specifically, both AP and transverse diameters of the FM were significantly larger in males than in females. ROC analyses further demonstrated that the FM-T parameter has high diagnostic accuracy for sex estimation (AUC = 0.992 ± 0.007, Accuracy = 0.971 ± 0.024), supporting the notion that FM measurements are reliable biometric indicators in forensic anthropology and cranial sex estimation. Similarly, a study on a Saudi population reported that both FM-AP and FM-T values were greater in males than in females [26]. At the same time, research on a Turkish population also identified higher mean sagittal and transverse diameters in males [17]. In a South Indian cohort, sagittal and transverse diameters predicted sex with 69.6% and 66.4% accuracy, respectively [27]. Despite these consistent findings, population-specific differences are also notable. Studies in Central European populations have also demonstrated significant dimorphism [28]. Such variability may reflect inherent genetic and morphological diversity, differences in measurement techniques, or variations in sample sizes. Given the anatomical and clinical relevance of the FM, establishing normative reference values across populations is essential. The FM forms the transitional region between the cranial base and the vertebral canal, housing vital structures such as the medulla oblongata and vertebral arteries. Defining its morphometric characteristics plays a critical role in the radiological evaluation of craniovertebral junction pathologies such as Chiari malformation and syringomyelia, as well as in determining safe surgical corridors during transcondylar approaches [17]. It has been reported that FM diameters are frequently reduced in cases of hypoplastic posterior cranial fossa, which may facilitate the development of tonsillar herniation [29]. Accordingly, our study provides high-accuracy FM morphometric data specific to the Turkish population, contributing valuable insight to both anthropological and clinical applications.

Examination of the morphometric characteristics of the IAO in our study revealed that both anteroposterior (RIAO-AP, LIAO-AP) and transverse (RIAO-T, LIAO-T) diameters were significantly larger in males than in females, confirming the presence of sexual dimorphism in this structure. ROC analysis further indicated that RIAO-T demonstrated exceptionally high discriminatory accuracy (AUC = 0.998 ± 0.004, Accuracy = 0.993 ± 0.014), suggesting that it may serve as a reliable biomarker in anthropometric evaluations. In addition, the ellipticity analysis showed that the IAO exhibited a more transversely elongated configuration in males compared to females, further supporting the presence of sex-related morphological differences in this structure. These findings reinforce the notion that not only linear but also shape-related parameters of the IAO exhibit sexual dimorphism, thereby broadening the scope of morphometric indicators useful for sex estimation. These findings are consistent with previous radiological studies on the Turkish population, which demonstrated significant sex-based differences in both horizontal and vertical diameters of the IAO using CT imaging [21]. However, contrasting results have been reported in dry temporal bone studies conducted on the same population, where no statistically significant sexual dimorphism was observed [22]. It has also been noted that these measurements may vary among different populations and ethnic groups [23,24,25]. This discrepancy may stem from methodological differences, including imaging technique, sample condition, and measurement precision, rather than true population-based variation. Our findings not only reinforce the relevance of sex-specific morphometric assessment in the Turkish population but also provide valuable anatomical insights that can inform preoperative planning and improve surgical safety. Importantly, variations in posterior cranial fossa morphology may also influence IAO anatomy; for instance, studies on PHACES cases have shown that hypoplastic posterior fossa structures are frequently accompanied by alterations in IAC/IAO configuration suggesting that posterior fossa underdevelopment can modify IOA morphology [30]. Clinically, detailed knowledge of IAO morphology—including its shape (circular, oval, or slit-like) and spatial relationships with the sigmoid sinus sulcus, superior petrosal sinus sulcus, and jugular foramen—is critical for neurosurgical and otologic procedures [31,32]. An oval or round IAO configuration may facilitate surgical maneuverability during suboccipital retrosigmoid approaches, whereas transversely elongated forms can restrict visualization [21]. Therefore, preoperative evaluation of IAO morphology and its topographic relationships via high-resolution CT or MRI is essential for optimizing surgical planning and minimizing operative complications [33,34].

In our study, morphometric analysis of the JF in the Turkish population revealed that both the anteroposterior and transverse diameters were larger bilaterally in males than in females. This finding demonstrates that the JF exhibits pronounced sexual dimorphism and may serve as a potential indicator for sex estimation from the skull. Indeed, the ROC analyses showed that the bilaterally obtained AUC values for these parameters were consistently high, ranging from 0.969 to 0.998, and the corresponding accuracy values varied between 0.905 and 0.993, indicating that these measurements can be used with high diagnostic accuracy to distinguish between sexes. The available literature supports these results. A study on a Thai population reported significantly larger anteroposterior and transverse diameters in males, emphasizing their utility in sex estimation [19]. However, noteworthy population-specific variations exist. For instance, an analysis in a Nigerian population found no significant sex differences in JF dimensions [35]. In contrast, in northeastern Brazil, males exhibited greater values in the anteromedial compartment, with no differences observed in the posterolateral compartment [36]. Similarly, research on the Indian population reported larger measurements on the right JF in males, while certain left-sided dimensions were greater in females [13]. These discrepancies underscore the influence of genetic, regional, and developmental factors on cranial base morphology.

The clinical relevance of such morphometric variability is substantial, given that the JF accommodates critical neurovascular structures, including the glossopharyngeal, vagus, and accessory nerves, as well as the sigmoid sinus and internal jugular vein. Morphological variations in and around the foramen may influence surgical corridors and pose risks during skull base and otologic procedures. JF stenosis is one of the most prominent findings of hypoplastic posterior cranial fossa [29]. In fact, individuals with a hypoplastic posterior cranial fossa, as seen in conditions like Chiari malformation or basilar invagination, often show a clear narrowing of the JF, and this narrowing has been noted to occur more prominently in females when these conditions appear on their own. The constriction becomes even more pronounced when both conditions are present together [37]. Similarly, in conditions characterized by posterior cranial fossa hypoplasia, such as achondroplasia, both the FM and the JF show marked reductions in size, which may clinically manifest as cervicomedullary compression and a potential risk of myelopathy [38]. Therefore, the population-specific morphometric data provided in our study make a meaningful contribution to the literature and strongly support the utility of JF measurements as reliable indicators for both anthropological sex estimation and surgical planning. Particularly for neurosurgical or craniovertebral junction procedures, measurements such as the JF–IAO distance or the distance between the JF and the anterior margin of the FM parameters included in the present study may be critical for defining safe operative corridors and avoiding injury to adjacent neurovascular structures.

Our study is unique in that it evaluates the relationship between the JF and IAO, as well as between the JF and FM anatomical metrics that have been scarcely examined in the existing literature. By doing so, it provides novel and clinically valuable morphometric references for skull base surgery. The significant sex differences identified between these structures, which align with prior findings, further support the validity of our measurements. Additionally, future studies incorporating the analysis of circularity ratios of these openings may enhance the reliability of morphometric data in this field.

While sex-related differences in FM and JF dimensions provide robust parameters for forensic sex estimation, the distance and diameter measurements obtained in our study contribute to establishing normative data specific to the Turkish population. Clinically, the skull base distances and diameters defined here may assist in delineating safe surgical boundaries, particularly in approaches targeting the craniovertebral junction and petroclival region. These morphometric references may help preserve neurovascular structures and optimize patient-specific surgical planning. Although the present study is limited to linear diameter and distance measurements, future investigations incorporating three-dimensional imaging techniques and larger sample sizes will provide a more comprehensive understanding of cranial morphometry from both anthropological and surgical perspectives.

## Figures and Tables

**Table 1:** Descriptive demographic characteristics of the cases.

	Female (*n* = 151)		Male (*n* = 153)		*p*
	Mean ± SD	Median (Min–Max)	Mean ± SD	Median (Min–Max)	
Age (year)	54.56 ± 20.69	55 (18–92)	51.74 ± 21.49	52 (18–93)	0.244
Height (cm)	162.24 ± 11.26	163 (143–186)	172.65 ± 11.03	173 (151–196)	<0.001
Head Circumference (cm)	54.82 ± 1.21	54 (51–58)	56.85 ± 1.66	56 (53–61)	<0.001

**Table 2:** Parameters and landmarks used for measurements.

Parameters	Landmarks	References
FM-AP	The maximum front and back length of the foramen magnum (basion–opisthion distance)	Vlajković et al. (2010) [16], Tellioglu et al. (2018) [17], Turamanlar et al. (2021) [18], Thunyacharoen et al. (2023) [19]
FM-T	The maximum width of the foramen magnum in transverse section	Vlajković et al. (2010) [16], Tellioglu et al. (2018) [17], Turamanlar et al. (2021) [18], Thunyacharoen et al. (2023) [19]
RJF-AP	The anteroposterior linear length measured between the most distant points along the endocranial opening of the foramen on the right side	Thunyacharoen et al. (2023) [19], Das et al. (2016) [13], Al-Redouan et al. (2022) [20]
RJF-T	The mediolateral linear length measured between the most distant points along the endocranial opening of the foramen on the right side	Thunyacharoen et al. (2023) [19], Das et al. (2016) [13], Al-Redouan et al. (2022) [20]
LJF-AP	The anteroposterior linear length measured between the most distant points along the endocranial opening of the foramen on the left side	Thunyacharoen et al. (2023) [19], Das et al. (2016) [13], Al-Redouan et al. (2022) [20]
LJF-T	The mediolateral linear length measured between the most distant points along the endocranial opening of the foramen on the left side	Thunyacharoen et al. (2023) [19], Das et al. (2016) [13], Al-Redouan et al. (2022) [20]
RIAO-AP	The linear distance extending from the most concave portion of the posterior lip of the right-sided opening to the most medial point of its anterior wall	Comert et al. (2024) [21], Sekerci et al. (2021) [22], Mamatha et al. (2019) [23], Papangelou (1972) [24], Marques et al. (2012) [25]
RIAO-T	The linear measurement connecting the upper and lower walls at the midpoint of the long axis of the right internal acoustic opening	Comert et al. (2024) [21], Sekerci et al. (2021) [22], Mamatha et al. (2019) [23], Papangelou (1972) [24], Marques et al. (2012) [25]
LIAO-AP	The linear distance extending from the most concave portion of the posterior lip of the left-sided opening to the most medial point of its anterior wall	Comert et al. (2024) [21], Sekerci et al. (2021) [22], Mamatha et al. (2019) [23], Papangelou (1972) [24], Marques et al. (2012) [25]
LIAO-T	The linear measurement connecting the upper and lower walls at the midpoint of the long axis of the left internal acoustic opening	Comert et al. (2024) [21], Sekerci et al. (2021) [22], Mamatha et al. (2019) [23], Papangelou (1972) [24], Marques et al. (2012) [25]

FM-AP, foramen magnum anteroposterior diameter; FM-T, foramen magnum transverse diameter; RJF-AP, right jugular foramen anteroposterior diameter; RJF-T, right jugular foramen transverse diameter; LJF-AP, left jugular foramen anteroposterior diameter; LJF-T, left jugular foramen transverse diameter; RIAO-AP, right internal acoustic opening anteroposterior diameter; RIAO-T, right internal acoustic opening transverse diameter; LIAO-AP, left internal acoustic opening anteroposterior diameter; LIAO-T, left internal acoustic opening transverse diameter.

**Table 3:** Intra-observer measurement precision values (TEM, rTEM, and R) for all morphometric parameters.

Parameters	TEM	rTEM (%)	R
FM-AP	0.004	0.01	0.987
FM-T	0.007	0.02	0.983
RJF-AP	0.007	0.05	0.973
LJF-AP	0.005	0.05	0.975
RJF-T	0.001	0.01	0.969
LJF-T	0.005	0.07	0.967
JJ	0.003	0.01	0.990
RJIAO	0.001	0.01	0.971
LJIAO	0.001	0.01	0.970
RIAOF	0.002	0.01	0.982
LIAOF	0.003	0.02	0.983
II	0.003	0.01	0.991
RJFM	0.004	0.03	0.981
LJFM	0.003	0.02	0.980
RIAO-AP	0.002	0.02	0.977
RIAO-T	0.001	0.01	0.968
LIAO-AP	0.004	0.04	0.977
LIAO-T	0.003	0.05	0.965

TEM, technical error of measurement; rTEM, relative TEM; R, reliability coefficient; FM-AP, foramen magnum anteroposterior diameter; FM-T, foramen magnum transverse diameter; RJF-AP, right jugular foramen anteroposterior diameter; RJF-T, right jugular foramen transverse diameter; LJF-AP, left jugular foramen anteroposterior diameter; LJF-T, left jugular foramen transverse diameter; RIAO-AP, right internal acoustic opening anteroposterior diameter; RIAO-T, right internal acoustic opening transverse diameter; LIAO-AP, left internal acoustic opening anteroposterior diameter; LIAO-T, left internal acoustic opening transverse diameter; JJ, distance between right and left jugular foramen; RJIAO/LJIAO, distance between jugular foramen and internal acoustic opening on the right/left; RIAOF/LIAOF, distance between internal acoustic opening and foramen magnum on the right/left; II, intermeatal distance; RJFM/LJFM, distance between jugular foramen and foramen magnum on the right/left.

**Table 4:** Morphometric measurements by sex (mm).

	Female		Male		*p*
	Mean ± SD	Median (Min–Max)	Mean ± SD	Median (Min–Max)	
FM-AP	34.45 ± 3.71	35.38 (28.25–43.54)	42.68 ± 2.40	42.26 (38.11–48.89)	<0.001
FM-T	32.64 ± 3.18	32.24 (27.02–40.19)	41.34 ± 2.27	40.94 (37.15–47.66)	<0.001
RJF-AP	10.20 ± 2.06	9.71 (7.18–15.75)	16.21 ± 1.18	16.60 (11.09–17.68)	<0.001
RJF-T	5.04 ± 1.35	4.97 (3.12–7.59)	9.36 ± 0.67	9.41 (6.28–12.55)	<0.001
LJF-AP	8.85 ± 1.93	8.38 (5.74–14.31)	14.27 ± 1.57	14.68 (9.14–16.09)	<0.001
LJF-T	4.65 ± 1.28	4.82 (2.75–7.18)	8.44 ± 0.65	8.49 (2.80–9.52)	<0.001
RIAO-AP	8.93 ± 2.02	8.39 (6.03–14.29)	14.84 ± 1.20	15.07 (9.61–16.62)	<0.001
RIAO-T	4.85 ± 1.34	4.81 (3.00–7.31)	9.23 ± 0.66	9.31 (6.05–12.57)	<0.001
LIAO-AP	7.56 ± 1.89	7.17 (4.41–13.12)	12.97 ± 1.53	13.43 (7.77–14.73)	<0.001
LIAO-T	4.55 ± 1.40	4.73 (2.57–8.42)	8.31 ± 0.84	8.32 (3.05–12.54)	<0.001
JJ	39.21 ± 3.55	38.25 (34.22–50.08)	46.88 ± 2.90	46.81 (42.07–60.72)	<0.001
RJIAO	5.84 ± 1.67	5.38 (3.42–10.01)	7.43 ± 1.79	7.46 (4.44–10.48)	<0.001
LJIAO	5.75 ± 1.67	5.24 (3.22–9.85)	7.32 ± 1.84	7.34 (4.19–10.44)	<0.001
RIAOF	16.35 ± 2.03	16.25 (11.93–21.02)	18.40 ± 1.73	18.31 (14.37–21.75)	<0.001
LIAOF	15.79 ± 2.12	15.84 (11.14–20.27)	17.66 ± 1.82	17.66 (12.92–21.22)	<0.001
II	44.50 ± 4.20	43.74 (37.14–59.44)	57.33 ± 5.38	55.10 (47.15–68.35)	<0.001
RJFM	15.59 ± 2.24	15.08 (11.84–20.23)	17.48 ± 2.03	18.23 (12.21–20.29)	<0.001
LJFM	15.21 ± 2.16	14.68 (11.80–20.21)	17.04 ± 2.01	17.64 (11.71–19.72)	<0.001

FM-AP, foramen magnum anteroposterior diameter; FM-T, foramen magnum transverse diameter; RJF-AP, right jugular foramen anteroposterior diameter; RJF-T, right jugular foramen transverse diameter; LJF-AP, left jugular foramen anteroposterior diameter; LJF-T, left jugular foramen transverse diameter; RIAO-AP, right internal acoustic opening anteroposterior diameter; RIAO-T, right internal acoustic opening transverse diameter; LIAO-AP, left internal acoustic opening anteroposterior diameter; LIAO-T, left internal acoustic opening transverse diameter; JJ, distance between right and left jugular foramen; RJIAO/LJIAO, distance between jugular foramen and internal acoustic opening on the right/left; RIAOF/LIAOF, distance between internal acoustic opening and foramen magnum on the right/left; II, intermeatal distance; RJFM/LJFM, distance between jugular foramen and foramen magnum on the right/left.

**Table 5:** Circularity (diameter ratio) values of cranial structures by sex.

	Female		Male		*p*
	Mean ± SD	Median (Min–Max)	Mean ± SD	Median (Min–Max)	
FM-E	0.92 ± 0.02	0.92 (0.89–0.99)	0.97 ± 0.03	0.97 (0.89–1.03)	<0.001
RJF-E	0.49 ± 0.07	0.49 (0.36–0.66)	0.58 ± 0.05	0.58 (0.50–0.76)	<0.001
LJF-E	0.52 ± 0.07	0.52 (0.40–0.69)	0.60 ± 0.08	0.60 (0.18–0.90)	<0.001
RIAO-E	0.54 ± 0.08	0.54 (0.40–0.74)	0.62 ± 0.06	0.62 (0.53–0.83)	<0.001
LIAO-E	0.61 ± 0.19	0.61 (0.38–1.71)	0.65 ± 0.10	0.65 (0.32–1.00)	<0.001

FM-E, foramen magnum ellipticity ratio; RJF-E, right jugular foramen ellipticity ratio; LJF-E, left jugular foramen ellipticity ratio; RIAO-E, right internal acoustic opening ellipticity ratio; LIAO-E, left internal acoustic opening ellipticity ratio.

**Table 6:** ROC analysis findings regarding sex estimation using morphometric measurements.

Parameters	AUC	Accuracy	*p*	Cut Off	Sensitivity	Specificity	Bias
FM-AP	0.957 ± 0.027	0.875 ± 0.062	<0.001	40.565	0.870 ± 0.096	0.874 ± 0.082	−0.004 ± 0.126
FM-T	0.992 ± 0.007	0.971 ± 0.024	<0.001	37.585	0.946 ± 0.044	0.994 ± 0.020	−0.048 ± 0.049
RJF-AP	0.983 ± 0.016	0.921 ± 0.042	<0.001	12.450	0.874 ± 0.085	0.965 ± 0.055	−0.091 ± 0.101
RJF-T	0.998 ± 0.004	0.993 ± 0.014	<0.001	7.920	1.000 ± 0.000	0.987 ± 0.027	0.013 ± 0.027
LJF-AP	0.969 ± 0.021	0.905 ± 0.062	<0.001	12.265	0.932 ± 0.061	0.871 ± 0.112	0.061 ± 0.127
LJF-T	0.992 ± 0.026	0.990 ± 0.016	<0.001	7.200	0.994 ± 0.019	0.985 ± 0.032	0.009 ± 0.037
JJ	0.944 ± 0.046	0.902 ± 0.046	<0.001	42.650	0.832 ± 0.090	0.970 ± 0.042	−0.138 ± 0.100
RJIAO	0.752 ± 0.047	0.691 ± 0.061	<0.001	5.460	0.541 ± 0.137	0.834 ± 0.088	−0.293 ± 0.163
LJIAO	0.743 ± 0.049	0.674 ± 0.047	<0.001	6.785	0.739 ± 0.088	0.600 ± 0.106	0.139 ± 0.138
RIAOF	0.791 ± 0.060	0.717 ± 0.053	<0.001	17.330	0.754 ± 0.095	0.676 ± 0.102	0.078 ± 0.139
LIAOF	0.754 ± 0.065	0.658 ± 0.051	<0.001	17.260	0.686 ± 0.187	0.624 ± 0.173	0.062 ± 0.255
II	0.984 ± 0.024	0.947 ± 0.038	<0.001	50.975	0.934 ± 0.074	0.959 ± 0.035	−0.025 ± 0.081
RJFM	0.740 ± 0.064	0.697 ± 0.064	<0.001	15.820	0.648 ± 0.083	0.747 ± 0.121	−0.099 ± 0.147
LJFM	0.746 ± 0.060	0.701 ± 0.061	<0.001	15.415	0.662 ± 0.089	0.741 ± 0.116	−0.079 ± 0.146
RIAO-AP	0.983 ± 0.015	0.916 ± 0.044	<0.001	11.895	0.868 ± 0.095	0.961 ± 0.057	−0.093 ± 0.111
RIAO-T	0.998 ± 0.004	0.993 ± 0.014	<0.001	7.725	1.000 ± 0.000	0.987 ± 0.027	0.013 ± 0.027
LIAO-AP	0.974 ± 0.018	0.915 ± 0.054	<0.001	11.115	0.939 ± 0.051	0.886 ± 0.095	0.053 ± 0.108
LIAO-T	0.975 ± 0.032	0.973 ± 0.031	<0.001	7.055	0.973 ± 0.035	0.970 ± 0.054	0.004 ± 0.064

AUC, area under the curve; FM-AP, anteroposterior diameter of foramen magnum; FM-T, transverse diameter of foramen magnum; RJF-AP/T, right jugular foramen anteroposterior/transverse diameter; LJF-AP/T, left jugular foramen anteroposterior/transverse diameter; RIAO-AP/T, right internal acoustic opening anteroposterior/transverse diameter; LIAO-AP/T, left internal acoustic opening anteroposterior/transverse diameter; JJ, distance between right and left jugular foramina; RJIAO/LJIAO, distance between jugular foramen and internal acoustic opening on the right/left; RIAOF/LIAOF, distance between internal acoustic opening and foramen magnum on the right/left; II, intermeatal distance; RJFM/LJFM, distance between jugular foramen and foramen magnum on the right/left.

## Data Availability

The data that support the findings of this study are not openly available due to ethical restrictions and are available from the corresponding author upon reasonable request.
